# The antibiofilm activity of selected substances used in oral health prophylaxis

**DOI:** 10.1186/s12903-022-02532-4

**Published:** 2022-11-17

**Authors:** R. Dudek-Wicher, A. F. Junka, P. Migdał, A. Korzeniowska-Kowal, A. Wzorek, M. Bartoszewicz

**Affiliations:** 1 Department of Pharmaceutical Microbiology and Parasitology, Faculty of Pharmacy, Medical University of Silesian Piasts in Wroclaw, 50-367 Wrocław, Poland; 2 Department of Environment, Hygiene and Animal Welfare, Faculty of Biology and Animal Science, Wroclaw University of Environmental and Life Sciences, Chełmońskiego 38C, 51-630 Wrocław, Poland; 3 Department of Immunology of Infectious Diseases, Polish Collection of Microorganisms (PCM), Hirszfeld Institute of Immunology and Experimental Therapy, Polish Academy of Sciences, Rudolfa Weigla 12, 53-114 Wroclaw, Poland

**Keywords:** Dysbiosis, Biofilm, Chlorhexidine, Cetylpyridinium chloride, Polyhexanide, Silver nanoparticles, Sulfonated phenolics, Coconut oil

## Abstract

Oral health is a window to a patient’s general well-being. Balance in oral microbiome functions is crucial for health maintenance. A state of oral dysbiosis may lead to a variety of local and systemic pathological conditions. The presence of dental plaque is related to the majority of oral infections. Proper oral hygiene is crucial and the most economic practice contributing to oral health prophylaxis. Aside from prophylactic treatments provided by dental practitioners, mouth rinses, containing antimicrobial agents, are one of the possible tools used for oral care. Our study was to determine whether available mouth rinses and selected products dedicated for professional use are efficient to eradicate biofilm formed by reference and clinical strains of *Streptococcus mutans*, *Streptococcus sanguinis, Streptococcus oralis, Streptococcus mitis, Staphylococcus aureus, Enterococcus faecalis, Lactobacillus rhamnosus* and *Candida albicans* on the surface of hydroxyapatite – major mineral component of a tooth. Therefore, such antimicrobials as chlorhexidine, cetylpyridine chloride, polyhexanide, silver nanoparticles, sulphonated phenolics, and natural antiplaque essential oils and coconut oil were analyzed. Applied experimental settings in in vitro models were designed to reflect accurately the recommended use of the tested substances, therefore four types of eradication procedure were conducted. Sialorrhea simulation was also performed to evaluate antibiofilm potential of diluted mouth rinses. Biofilm was investigated with quantitative method where absorbance values were measured. Statistical differences were assessed using the Kruskal–Wallis test with a post-hoc Dunnett’s analysis. Results have shown that biofilms displayed a diversified sensitivity to the tested antimicrobials. The highest antibiofilm activity was detected for cetylpyridine chloride while the lowest for chlorhexidine. However the differences in *E. faecalis* biofilm reduction observed after the use of these two compounds were not statistically significant (*p* > 0.05), whereas all observed differences in *S. aureus* survival after exposure to the examined antimicrobial agents were statistically significant (*p* < 0.5). The PHMB, both in standard and in sialorrhea simulated conditions had the highest potential against streptococci. The coconut oil reduced *C. albicans* fungus biofilm by 65.48% but low eradication level was observed in case of bacterial biofilms. The dehydrating mechanism of action of sulfonated phenolics turned out to be ineffective against streptococcal biofilm which in turn was effectively eradicated by silver nanoparticles. The implementation of Antibiofilm Dressing's Activity Measurement method allowed to observe strain-related differences in terms of antimicrobial sensitivity. The obtained results may be introduced in everyday out-patient dental plaque prophylaxis as well as clinical environment.

**Supplementary Information:**

The online version contains supplementary material available at 10.1186/s12903-022-02532-4.

## Introduction

The pivotal role of oral microbiome in human health and disease is widely recognized. The disruption of the oral microbiome leads to dysbiosis resulting in the development of numerous local pathological conditions including dental caries, periodontal diseases or oral cancers [1, 2]. According to data provided by the World Health Organization, the oral diseases affect nearly 3.5 billion people globally and they are classified as the most common diseases of chronic character [3, 4]. Apart from dental caries, which remains the most prevalent dental problem in childhood, periodontitis affects 5–15% members of population and oral cancer is the eighth most common cancer worldwide [5, 6]. The persistent oral infections may contribute to the development of such systemic diseases as cardiovascular disease, diabetes mellitus, rheumatoid arthritis, and Alzheimer’s disease [7]. Moreover, periodontitis may be associated with a higher risk of complications from COVID-19, including ICU admission and death [8].

It is well-established that the presence of dental plaque (microbial biofilm of adhered, multi-cellular structure embedded within a protective matrix) correlates with incidences of oral diseases [9].

The oral health prophylaxis struggles with biofilm localized on teeth surface. The dental practitioners provide professional prophylactic treatments to combat dental plaque. Unfortunately, these procedures are not available for the majority of patients due to the high economic cost [10–12]. Therefore, the preservation of oral hygiene is the key to maintaining oral health. One of the most cost-effective approaches is the application of mouth rinses containing such antimicrobials as chlorhexidine (CHX), cetylpyridine chloride (CPC), polyhexanide (PHMB) or essential oils (EO) [13]. There is also evidence indicating coconut oil as a good option to keep proper oral hygiene [14].

Therefore, the goal of this study was to assess the antibiofilm efficacy in vitro of 4 mouth rinses and coconut oil as well as silver nanoparticles (AgNP) solution and sulphonated phenolics gel (HY), both designated for dental professional use. Above-mentioned products were applied against biofilms formed on hydroxyapatite by the pathogens that may contribute to oral diseases, including *Streptococcus mutans*, *Streptococcus sanguinis, Streptococcus oralis, Streptococcus mitis, Staphylococcus aureus, Enterococcus faecalis, Lactobacillus rhamnosus* and *Candida albicans* using a spectrum of adequate techniques for biofilm measurement [15].

## Materials and methods

Into the research, 8 reference strains from American Type Culture Collection (ATCC) and Polish Collection of Microorganisms (PCM) and 95 clinical strains listed in Appendix Table 1.were applied.

### Biofilm culturing

From the 24-h culture of each test organism, a suspension of 1 MacFarland turbidity was prepared and diluted 1000-fold to 1 × 10^5^ CFU/ml in modified artificial saliva (0,9% NaCl – 90%; MHB – 5%,10% mucin—5%)[16]. Sterile hydroxyapatite (HA) discs (Ø 8.5 mm), manufactured as described by Junka et al., were placed in the wells of a 24-well plate [17]. Then, 2 mL of each of the prepared microbial suspensions were applied to each well in 6 replications. Plates were incubated in 37 °C/ 5% CO_2_, 24 h. Afterward, biofilm coated HA discs were transferred to new 24-well plates and subsequently, eradication procedures were performed. The 95 clinical strains used in this investigation were chosen based on biofilm producing capacities, determined in crystal violet assay.

### Biofilm eradication

#### Common use products: mouthrinses

Mouthrinses were applied against 8 reference and 95 clinical strains (Appendix Table 1).

The HA discs covered with tested biofilms were rinsed with 2 mL of the assessed mouthrinses containing different antimicrobial agent. Rinsing (contact) time was selected according to the manufacturer's recommendations:

- Chlorhexidine (CHX): 60 s,
- Polyhexanide (PHMB): 30 s,
- Cetylpyridinium chloride (CPC): 30 s,
- Cetylpyridinium chloride with Essential Oil Blend (CPC-EO): 60 s.

As a negative control, 2 mL of 0.9% NaCl was used for 30 or 60 s, respectively.

The tests were performed in 6 replicates.

### Sialorrhea simulation

All tested mouthrinses were diluted 1:1 with artificial saliva and applied as described above against 8 reference strains.

#### Professional use products: silver nanoparticles solution (AgNP) and sulfonated phenolics gel (HY)

The solution of AgNP was applied against 8 reference strains and 22 clinical strains while HY gel was applied against 8 reference strains (Appendix Table 1).

**Fig. 1:**
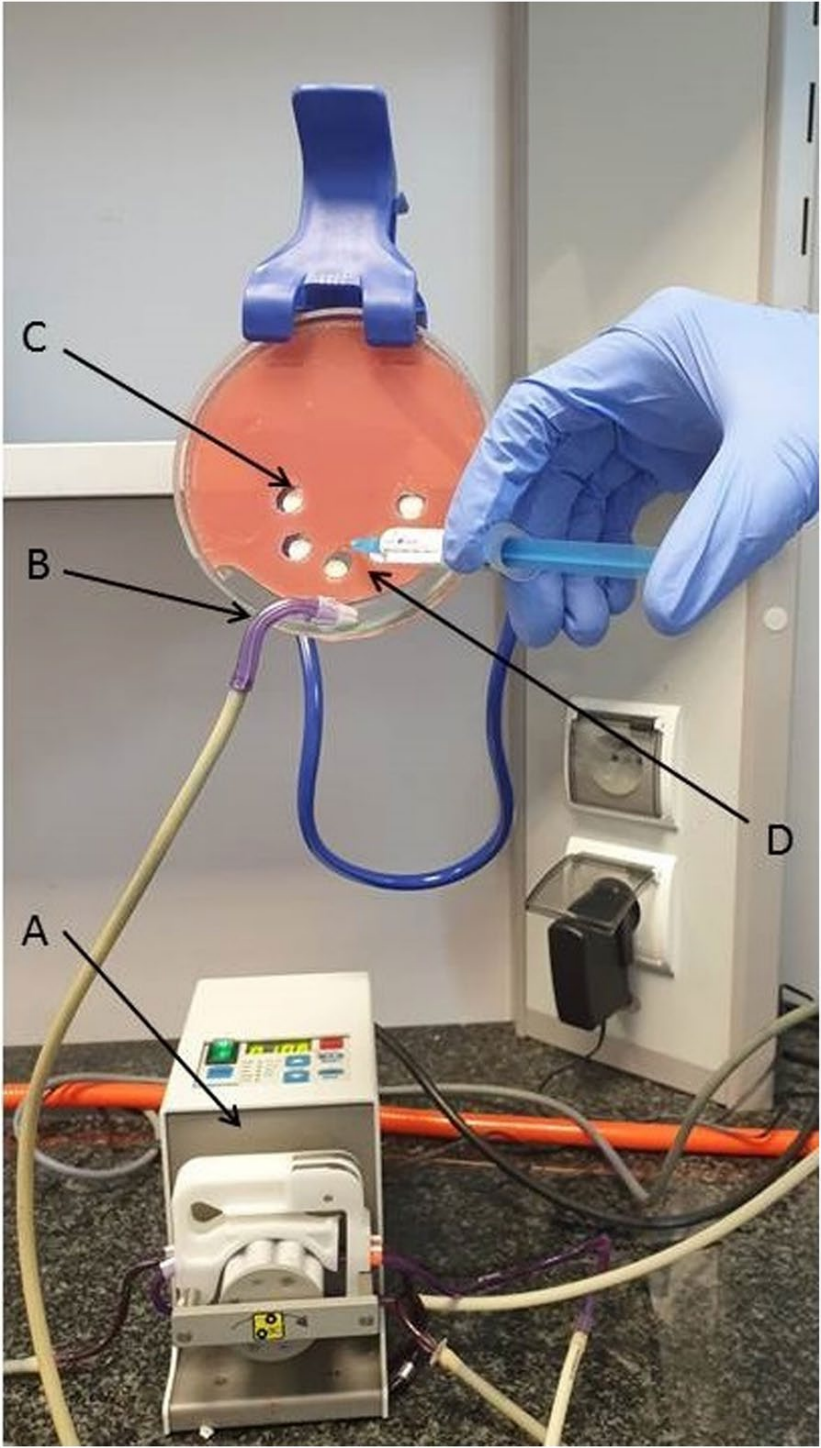
Rinsing of biofilm-coated hydroxyapatite discs placed in the artificial gingiva. **A**-peristaltic pump, **B**- saliva ejector placed in a depression simulating the sublingual area, **C**-HA disc covered with biofilm surrounded by artificial gingiva. **D**- Rinsing the pockets with silver nanoparticles solution. For the picture clarity, the whole experimental setting is shown outside an aseptic laminar chamber

HA discs covered with tested biofilms were placed in the holes made in TSA (Tryptone Soy Agar*;* Biomaxima, Lublin, Poland) to simulate gingival pockets (Appendix Fig. 1). Afterwards, the discs were rinsed with 1.5 mL of the investigated AgNP solution (pace 0.5 mL/ second). As a negative control 1.5 mL of 0.9% NaCl (pace 0.5 mL /second) was used. The fluid excess was aspirated with a dental saliva ejector connected to a peristaltic pump (Regli Digital MS2/12, Ismatec, Wertheim, Germany). This procedure was performed to simulate the rinsing of gingival pocket performed by dental professionals (Fig. 1).

**Fig. 2:**
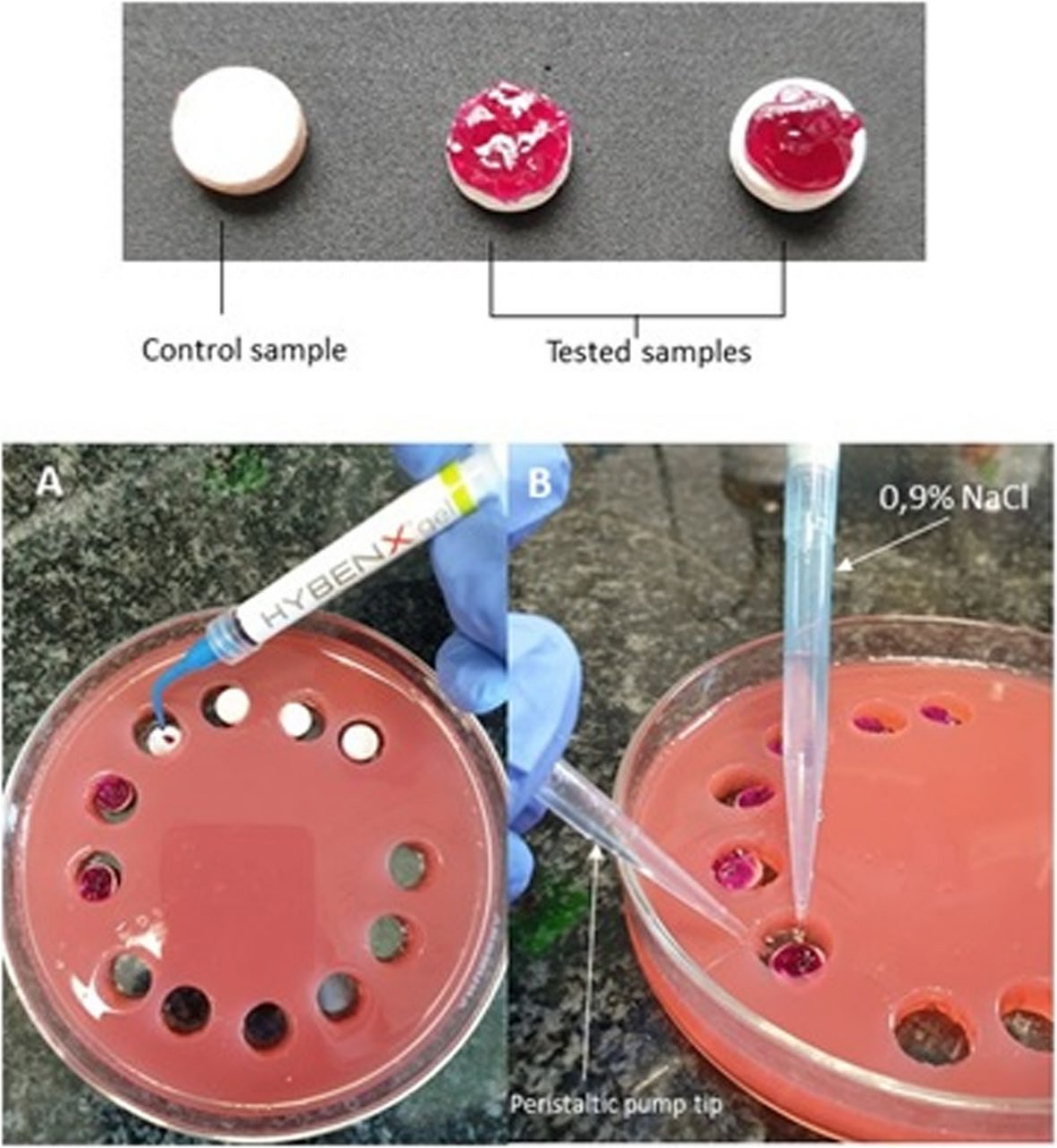
HY testing method and application mode. **A**—HY gel application on surfaces of HA discs covered with biofilm. **B** – rinsing HY gel from the surface with simultaneous suction of excess liquid

In the case of sulfonated phenolics gel, 0.3 mL of the HY gel was applied onto the biofilm coated discs for 30 s, then rinsed with 1 mL of 0.9% NaCl (Fig. 2). As a control, rinsing with 1 mL of 0.9% NaCl of investigated biofilms was performed. The excess of fluids was drained with a tip attached to peristaltic pump (Regli Digital MS2/12, Ismatec, Wertheim, Germany). All tests were performed in 6 replications.

The ingredients of all tested products are presented in Appendix Table 2.

#### Natural product: coconut oil

Coconut oil (Bio Planete, Bram, France) was applied against 8 reference strains.

The HA discs covered with tested biofilms were flooded with 1.5 mL of warm (37 °C) coconut oil. The control discs were flooded with 1.5 mL of 0.9% NaCl. The plates were shaken on a microtitrate plate shaker (IKA Schuttler MTS 4) (37 °C/ 5% CO_2_) for 20 min (300 RMP) to simulate oil pulling performed by a patient. All tests were performed in 6 replicates.

### Measurement of biofilm eradication activity

Both test and control samples from each part of investigation were stained. For this purpose, 1.5 mL of 0.1% TTC (2,3,5-triphenyl-tetrazolium chloride, AppliChem Gmbh, Damstadt, Germany) in TSB ( Tryptone Soya Broth, BTL, Warszawa, Poland) were added to wells with *Staphylococcus* spp, *Enterococcus* spp. and *Candida* spp. biofilms; 0.1% TTC in MRS (Man, Rogosa, Sharpe, Biomaxima, Lublin, Poland) for *Lactobacillus* spp. biofilms; and 0.1% TTC in BHI (Brain Heart Infusion, Franklin Lakes, New Jersey, U.S.A) for *Streptococcus* spp. biofilms. All 24-well plates with control and tested samples immersed in above-described solutions were incubated in 37 °C/ 5% CO_2_ for 4 h. During this time, TTC was metabolized by tested microorganisms to red 3,5-triphenylformazan. Then, the discs were transferred to new wells in 24-well plates. Each disc was flooded with 1.5 mL of 100% methanol to dissolved produced 3,5-triphenylformazan. The plates were shaken (400 RPM) on microtitrate plate shaker (IKA Schuttler MTS 4) for 20 min at 37 °C. Afterwards, 3 samples of 200 µl from each well were transferred to wells on 96-well plates. The absorbance measurement was performed at a wavelength of 490 nm using Scan Go spectrometer (ThermoScientific™ Multiskan™ GO Microplate Spectrophotometer, Waltham, MA, USA).

The eradication rate was calculated using the formula:

$$\%\mathrm{E }= \left({\mathrm{Ab}}_{\mathrm{1,2},3\dots }/\mathrm{Ak}\right) * 100\mathrm{\%}$$

E- Eradication.

Ab_1,2,3…._ = Absorbance of the sample no.1,2,3 etc.

Ak = Average absorbance of control samples (positive control).

The example of HA discs covered with TTC—stained biofilm is presented in Appendix Fig. 4.

### Biofilm eradication measured with A.D.A.M method (Antibiofilm Dressing's Activity Measurement)

The aim of the experiment was the semi-quantitative evaluation of the number of viable biofilm-forming cells on the surface of hydroxyapatite discs in artificial saliva after applying biocellulose (BC) dressings saturated with antimicrobial agent. BC was obtained and purified according to the procedure described by Dydak et al. [18].

Based on the results obtained in the eradication section, the most effective antimicrobial agents were selected for this part of the experiment.

This method was performed according to the protocol presented by Junka et al. [19]. Hydroxyapatite (HA) discs were coated with biofilm of 8 reference strains. For this purpose, the HA discs were placed in a 24-well plate and flooded with 2 mL of a microbial suspension of 1 × 10^5^ cells/mL. The incubation was carried out at 37 °C/5%CO_2_ for 24 h. Simultaneously, biocellulose discs were placed in the wells of a 24-well plate, covered with 2 mL of selected substances: AgNP, CPC, CPC-EO, PHMB and incubated in a fridge at 4 °C (laboratory freezer CHL 5 BASIC, POL-EKO) for 24 h. Biocellulose for negative control was incubated in 2 mL 0,9% NaCl. Biocellulose structure is presented in Appendix Fig. 2 of Supplementary materials. The next day, agar tunnels were made in the wells of the 24-well plate. Each HA disc coated with biofilm was placed at the bottom of the tunnel and flooded cautiously with artificial saliva. At the top of the tunnel, biocellulose saturated with tested substances was placed and covered with a polystyrene disc. Plates were incubated in 37 °C for 24 h. Afterwards, HA discs were transferred to new 24-well plates and measurement of biofilm eradication activity was performed according to above-described procedure.

The tests were performed in 6 replications. The experimental setting of the A.D.A.M method is presented in Appendix Fig. 3 of Supplementary materials.

### Biofilm morphology imaging

The structure of HA discs and biofilms formed by reference strains of *S. mutans* and *L. rhamnosus* on HA discs before and after eradication with PHMB and CPC, was analyzed using SEM (Auriga 60, Zeiss, Jena, Germany).

Biofilm coated HA discs (control and tested samples) were fixed in glutaraldehyde (Chempur, Piekary Slaskie, Poland) and subjected to the sputtering with Au/Pd (60:40) using a high vacuum coater (EM ACE600, Leicasputter, Leica Microsystems, Wetzlar, Germany).

### Statistical analysis

GraphPadPrism 8.0.1 (GraphPad Software, Inc., San Diego, California, USA) was used for statistical studies. The normality of the distribution was assessed using the D'Agostino-Pearson omnibus test. As all the values were not characterized by a normal distribution, Statistical differences were assessed using the Kruskal–Wallis test with a post-hoc Dunnett’s analysis. The results were considered significant at *p* < 0.0001.

**Fig. 3:**
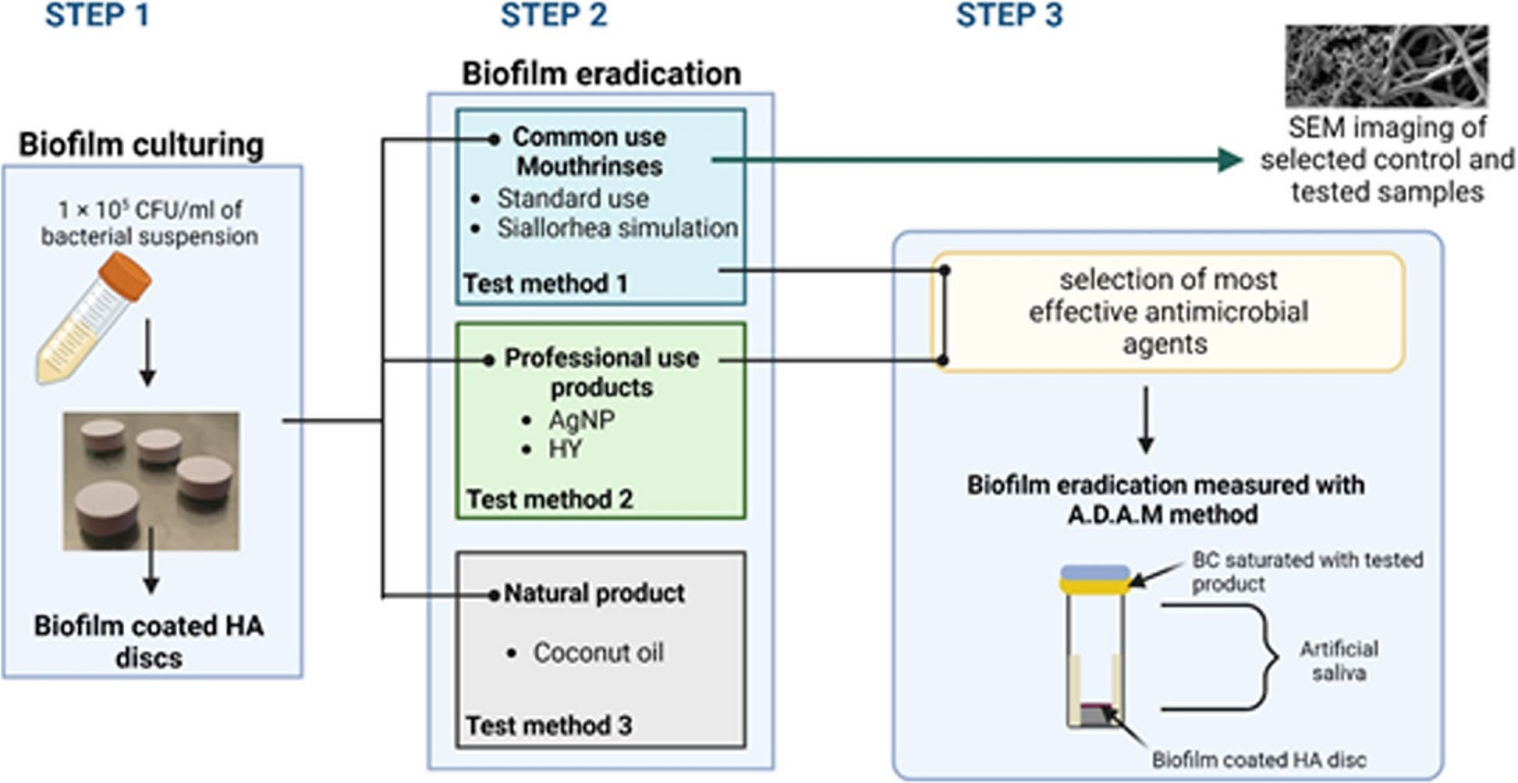
Graphical summary of the methodology

The summary of all methods applied is presented in Fig. 3.

## Results

### Biofilm eradication: mouthrinses

**Fig. 4:**
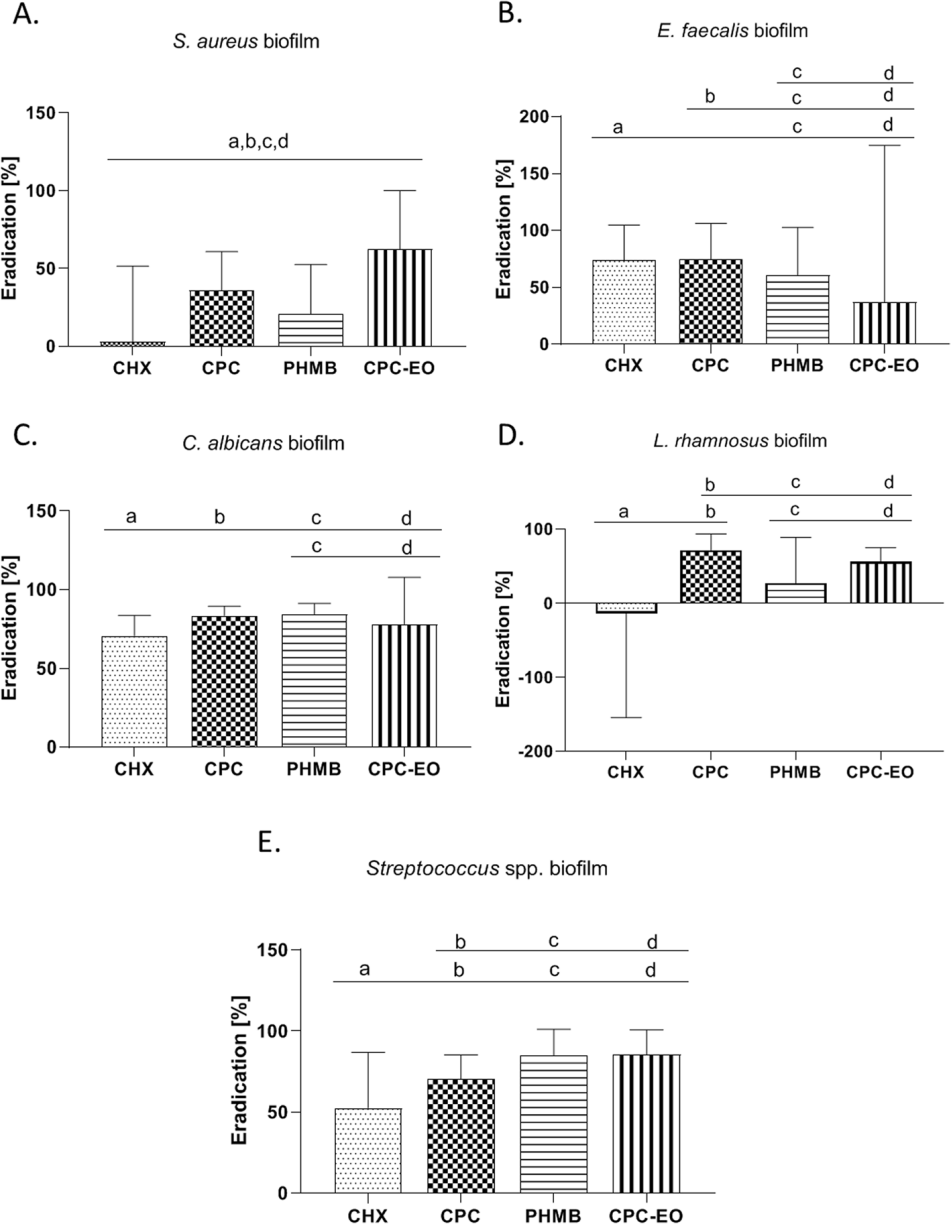
Reduction of *S. aureus* (**A**), *E. faecalis* (**B**), *C. albicans* (**C**), *L. rhamnosus* (**D**), *Streptococcus* spp. **E** biofilm from the HA surface after the use of: CHX (chlorhexidine), CPC (cetylpyridinium chloride), PHMB (polyhexanide), CPC-EO (cetylpyridinium chloride with EOs blend). a, b, c, d —significant differences between tested mouthrinses

Of the examined antimicrobial agents, a complex of CPC-EO indicated the highest activity towards *S. aureus* biofilm. It has displayed 59.39% greater antibiofilm potential than CHX. The CPC was the second most effective against *S. aureus* biofilm among tested agents (Fig. 4A). All observed differences in *S. aureus* survival after exposure to the examined antimicrobial agents were statistically significant (*p* > 0.0001). However, it should be noted that the standard deviations marked on the chart (Fig. 4A) were of relatively high values, which indicates diverse tolerance patterns of particular tested *S. aureus* strains against individual antimicrobial agent.

The strongest reduction of *E. faecalis* biofilm (Fig. 4B) was observed after the use of CPC (50.92%) and CHX (48.96%). The differences in biofilm reduction observed after the use of these two compounds were not statistically significant. CPC-EO solution showed the weakest ability to reduce *E. faecalis* biofilm.

PHMB and CPC showed the strongest eradication potential of all test substances against *C. albicans* biofilm—83.57%, 84.15% respectively (Fig. 4C). Differences in the effectiveness between these two formulations were not statistically significant. PHMB and CPC activity was in turn significantly higher compared to CHX and CPC-EO (*p* > 0.0001). The weakest average reduction of *C. albicans* biofilm (equals of 70.64%), was observed after the use of CHX. Similarly as it was noticed for *E. faecalis* biofilm, the high value of standard deviation from the mean was noticed after the use of CPC-EO also in the case of *C. albicans* biofilm (Fig. 4B, C).

The most active substance towards *L. rhamnosus* biofilm was CPC (biofilm reduction by 76.33%), while the lowest activity in relation to *L. rhamnosus* biofilm was observed after CHX use (Fig. 4D). The level of biofilm eradication after CHX application was significantly lower compared to the results obtained after eradication performed with use of CPC, PHMB, CPC-EO (*p* > 0.0001). The CPC and CPC-EO showed significantly higher antibiofilm efficacy than PHMB. CPC activity was significantly higher than CPC-EO (*p* > 0.0001) (Fig. 4D).

The highest effectiveness against *Streptococcus* spp. biofilm was observed in the case of PHMB (reduction of biofilm 84.20%) and CPC-EO (reduction of biofilm 85.47%) (Fig. 4E). The differences in biofilm eradication efficacy between these two formulations were statistically insignificant. The CPC antibiofilm activity (biofilm reduction by 70.46%) was in turn significantly higher compared to CHX activity (*p* > 0.0001); (biofilm reduction 52.06%)(Fig. 4E).

### Sialorrhea simulation

**Fig. 5:**
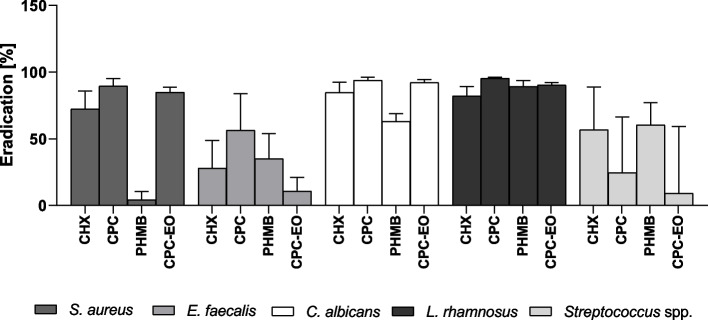
Reduction of biofilm formed by 8 reference strains on the HA surface after use of tested compounds diluted 1:1 with AS: CHX (chlorhexidine), CPC (cetylpyridinium chloride), PHMB (polyhexanide), CPC-EO (cetylpyridinium chloride with EOs blend)

The data shown in the Fig. 5 indicates highly diversified biofilm tolerance patterns expressed by individual species towards particular antimicrobial agents. For example, a solution of CPC-EO has shown the highest activity against *S. aureus* and *C. albicans* biofilms and the lowest activity toward *E. faecalis* or *Streptococcus* spp. biofilms. CPC had the highest eradication potential in relation to *S. aureus, E. faecalis, C. albicans* and L. *rhamnosus* biofilms. In turn, the highest efficacy against biofilm formed by the S*treptococci* was demonstrated by PHMB.

**Fig. 6:**
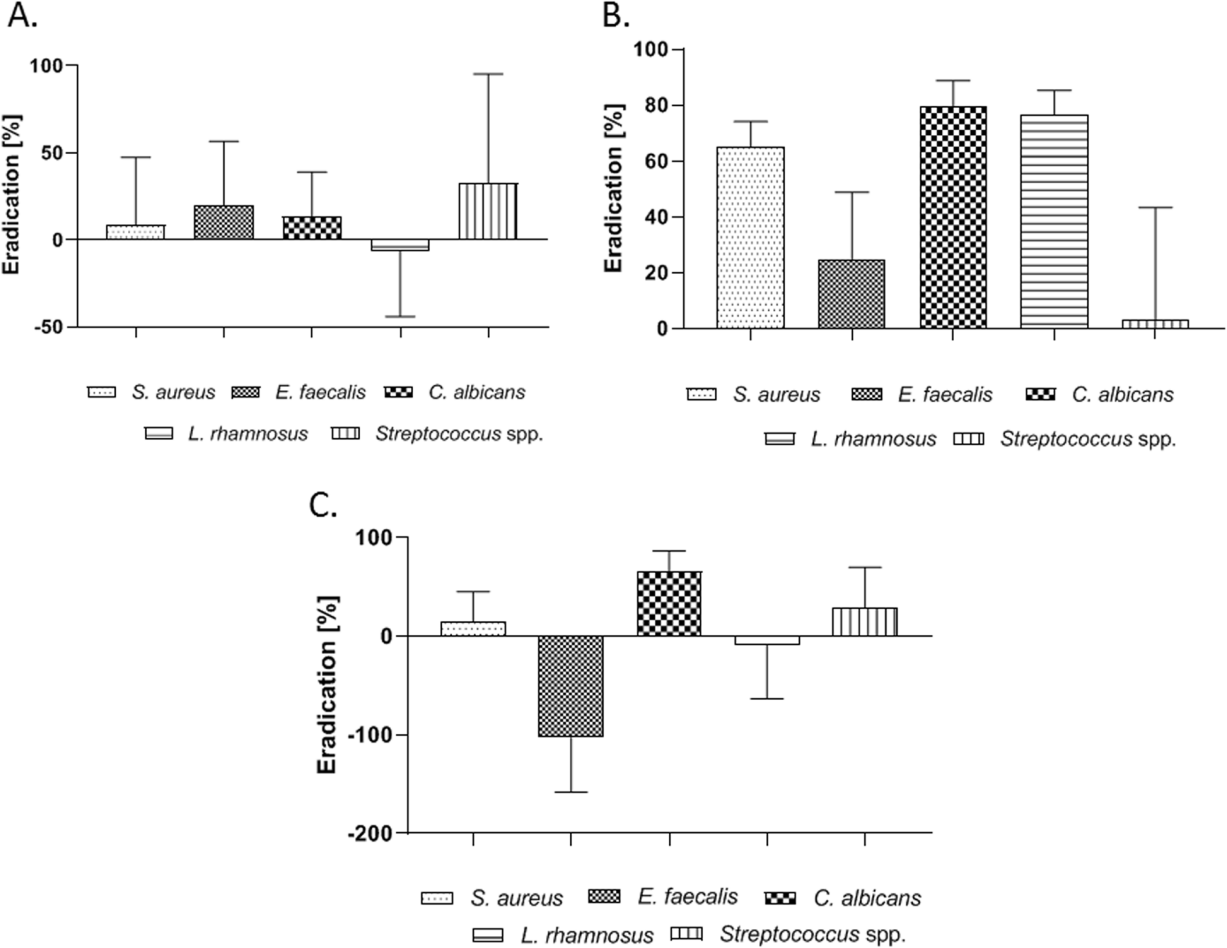
Reduction of biofilm formed on HA surface after applying: AgNP solution (**A**), HY gel (**B**) and after coconut oil pulling simulation (**C**)

The use of AgNP solution led to a strong biofilm eradication formed by *Streptococcus* spp. (Fig. 6A) The level of this eradication was significantly stronger (37.28%) compared to the eradication level observed in case of *S. aureus*, *C. albicans* and *L. rhamnosus* biofilms. There was no statistical significance in *E. faecalis* biofilm reduction. AgNP was the least effective against *L rhamnosus* biofilm.

Figure 6B presents the results of biofilm eradication with HY gel. The use of sulphonated phenols gel reflected in a significantly more effective eradication of *C. albicans* (79.39%) and *L. rhamnosus* (76.61%) biofilm than *E. faecalis, S. aureus* and *Streptococcus* spp. (*p* > 0.0001). However it should be noticed that *S. aureus* biofilm showed a significantly higher sensitivity to sulfonated phenols (65.21% eradication) than biofilms formed by *E. faecalis* and *Streptococcus* spp.. The results presented in Fig. 6C indicate that coconut oil reduced *C. albicans* biofilm by 65.48%. The lowest eradication level was observed in case of *E. faecalis and L. rhamnosus* biofilms. Comparable eradication level was observed for *S. aureus* and *Streptococcus* spp. biofilms, 15.04% and 28.46% respectively.

**Fig. 7:**
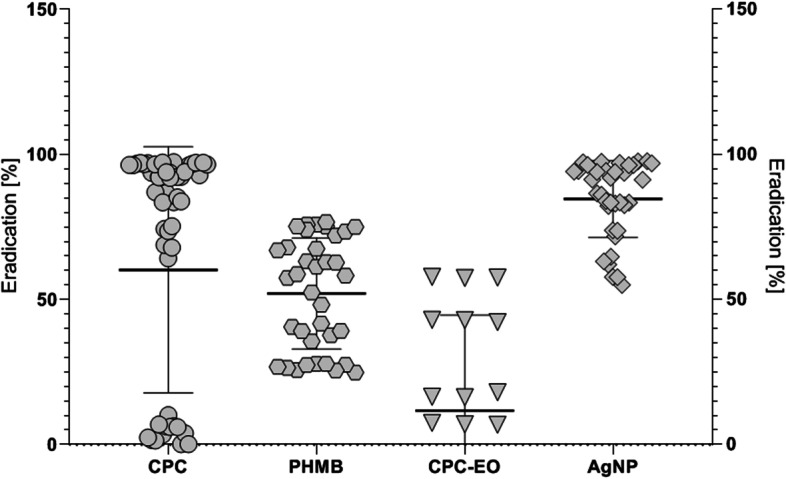
Reduction of biofilm formed on the HA surface measured by the A.D.A.M method

The results of A.D.A.M. method have shown that AgNP has displayed the highest activity against the tested microorganisms—*S. oralis* and *S. sanguinis* (Fig. 7). A similar sensitivity was demonstrated by *S. mutans* and *C. albicans* after PHMB application. The antibiofilm potential of CPC is strain-dependent. The lowest susceptibility to the antimicrobial agent activity was observed in case of *E. faecalis* biofilm against which a CPC-EO solution was applied.

### Biofilm SEM visualization

Before biofilm eradication was performed, the presence of biofilm, formed on HA surface, was verified by means of SEM.

**Fig. 8:**
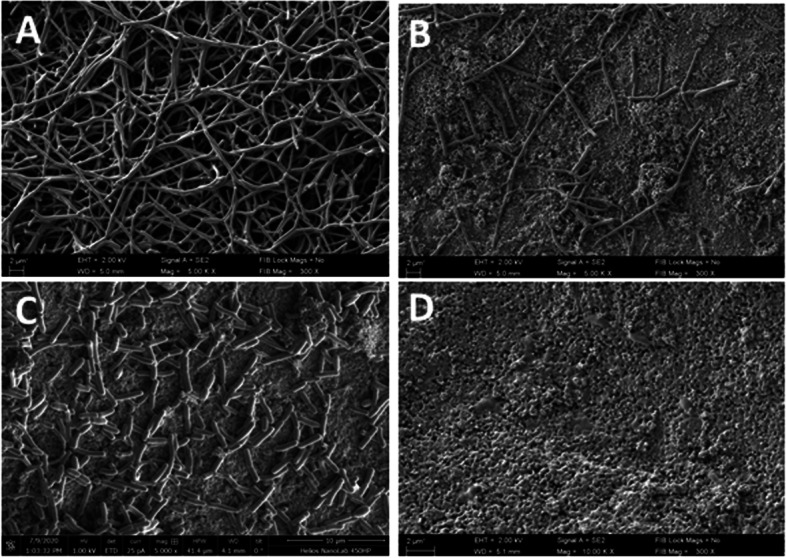
**A**—*L. rhamnosus* biofilm on HA discs (control sample**,** magnification equal 5000 ×), **B**—*L. rhamnosus* biofilm after eradiation with PHMB (magnification equal 5000 ×), **C**—*L. rhamnosus* biofilm after eradiation with CPC (magnification equal 5000 ×), **D**—Porous structure of HA discs which imitates the mineral component of the tooth surface ( magnification equal 10 000 ×)

In Fig. 8A, the structure of *L. rhamnosus* biofilm, formed on HA disc, is presented. The structure of the biofilm is highly organized. Figure 8B was taken after *L. rhamnosus* biofilm eradication, performed with PHMB. The exposed places on hydroxyapatite disc and visible disorganization of the biofilm structure implicate the antimicrobial effect of PHMB. A similar phenomenon of reduction the biofilm biomass can be seen in Fig. 8C, which shows the biofilm remaining on the surface of hydroxyapatite after using the CPC mouthrinse.

**Fig. 9:**
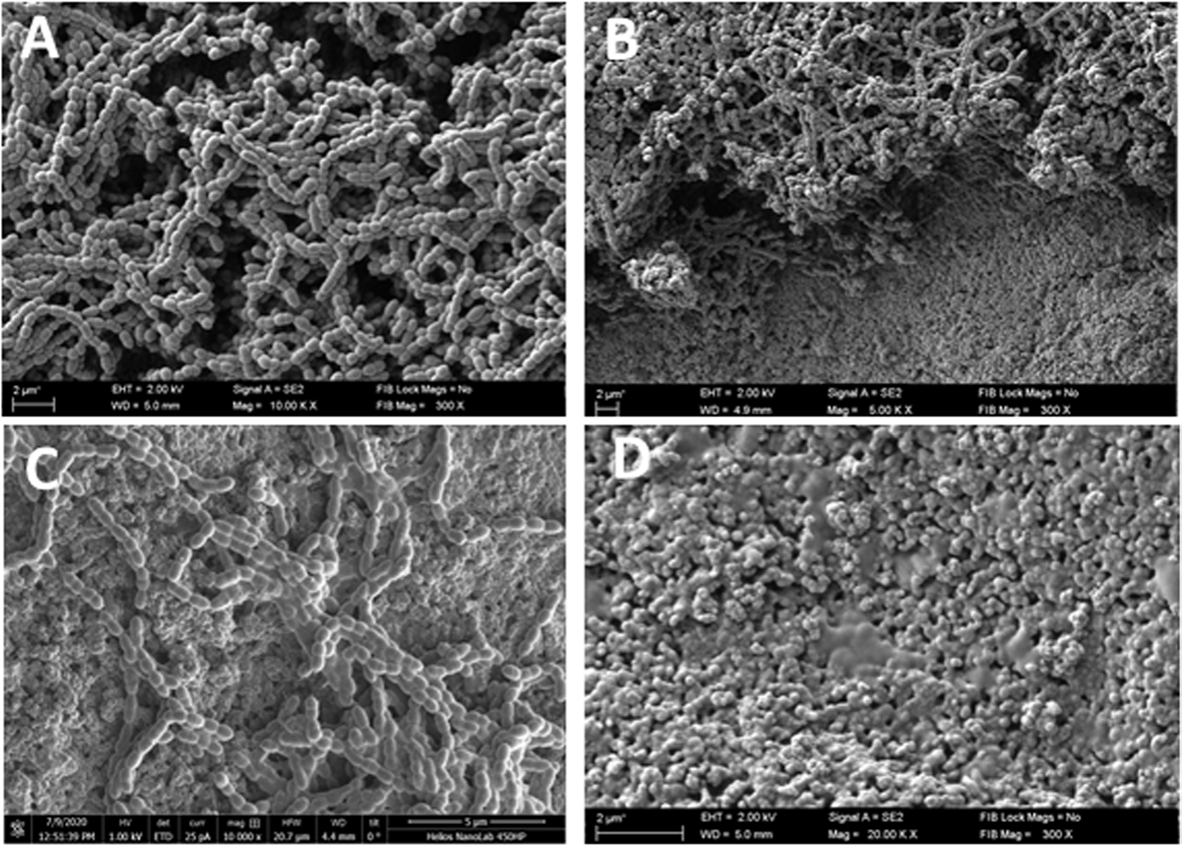
**A**—*S. mutans* biofilm on HA discs (control sample**,** magnification equal 10,000 ×), **B**—*S. mutans* biofilm after eradiation with PHMB (magnification equal 5000 ×), **C**—*S. mutans* biofilm after eradiation with CPC (magnification equal 10 000 ×).** D**—Porous structure of HA discs which imitates the mineral component of the tooth surface ( magnification equal 20 000 ×)

In turn in Fig. 9A, B and C the structure of *S. mutans* biofilm on HA discs is presented. Figure 9A demonstrates dense, organized in cellular chains, biofilm structure, distinctive for this species. Figure 9B shows the eradication effect of *S. mutans* biofilm using a polihexanidine (PHMB) mouthrinse. Figure 9C shows the effect of *S. mutans* biofilm biomass reduction after using a CPC-containing mouthrinse.

The porous structure of hydroxyapatite disc showed in two different magnification in Figs. 8D and 9D, imitates the mineral component of the tooth surface. Mineral microspheres that build the hydroxyapatite disc favor the adhesion and multiplication of microbial cells.

In Appendix Fig. 5 an example of a multispecies biofilm dominant in the oral environment is demonstrated. For this purpose biofilm formed by two species: *S. mutans* and *L. rhamnosus* has been cultured.

## Discussion

The effective prophylaxis is a crucial strategy for dental plaque development control and maintaining the health of the oral cavity. Preventive actions are based on mechanical or chemical dental biofilm removal from the tooth surface.

The application of CHX, CPC, PHMB, CPC/EO, AgNP, HY belongs to the chemical type of biofilm removal. CHX is still considered a “gold standard” in oral antisepsis. Nevertheless, it promotes mineral uptake into the biofilm and contribute to dental calculus formation [20]. All analysis described in this study were performed with coherent spectrum of techniques as presented in Fig. 3. The CHX mechanism of action makes it effective against planktonic cells and prevents biofilm formation in the early stages of its development. In our study, the weak anti-biofilm activity against mature biofilm observed in case of all tested biofilms (Fig. 4A–E), except *E. faecalis* biofilm, may result from a poor penetration of CHX molecule through the extracellular polymeric matrix (EPM*).* It was already proven that in mature biofilm, the negatively charged components of EPM including extracellular DNA (eDNA) may hinder the diffusion of positively charged CHX molecules [21]. Cieplik et al. has reported presence of efflux pumps which are membrane proteins containing multiple transmembrane domains that allow to eliminate the antimicrobials from cytoplasm. These proteins could increase tolerance of tested strains toward CHX [22]. Relatively high anti-biofilm activity of CHX against *E. faecalis* and *C. albicans* biofilm may suggest that EPM produced by this species contain less negatively charged eDNA than EPM of other species or cells are not equipped with efflux pump [23]. Anti-biofilm activity of CHX against *C. albicans* biofilms was described also by Alvendal et.al.[24].

Chlorobutanol presented in CHX formula is considered as antimicrobial preservative used in multi-dose drug formulations, however its antibiofilm potential is not evaluated yet [25, 26]. Based on antimicrobial mechanisms of action, chlorobutanol may have a synergistic effect with CHX against tested biofilms. Ruiz et al. experiment suggest high antimicrobial potency of CHX, however, they investigated planktonic forms and the contact time of the rinse with the microorganism ranged from 18-48 h [27]. Babu et al. has described the high efficiency of CHX against biofilms on hydroxyapatite discs after 10 min of contact time [28]. Such a long time of contact with microorganisms is far from the real conditions of use. In this study contact time equals 1 min, therefore, weak anti-biofilm effect was observed. It can be then concluded that the length of the exposure time, translate into antibiofilm outcomes [29].

It was estimated that PHMB was active against all tested biofilms. The highest level of biofilm removal (eradication) was observed against *Streptococcus* spp and *C. albicans* biofilms. PHMB was also more effective than CHX in eradication of mature biofilm as it was shown already by another research team [30]. The results presented in our investigation, support this conclusion. According to other authors, the PHMB, once inside the cell, can effectively "bind" to DNA through extensive interactions with DNA phosphate backbone, which can potentially block the DNA replication process [31]. The betaine, surfactant molecule present in the applied mouthrinse solution, reduces surface tension and may facilitate penetration of PHMB through the biofilm structure, its binding to the phospholipids of cell membrane and cell infiltration, finally [32]. This may be the reason of higher effectiveness of PHMB than CHX against the tested biofilms. PHMB was also the most effective when diluted with artificial saliva (Fig. 5) but only against *Streptococcus* spp.

The CPC’s high antibiofilm activity against all tested species was revealed in our study (Fig. 4A-E). Such a phenomenon was also observed by Latimer et al. [33]. CPC also proved to be the most effective against *S. aureus, E. faecalis, C. albicans, L. rhamnosus* biofilms in the sialorrhea simulation test where mouthrinses were diluted 1: 1 with artificial saliva (Fig. 5). The reason for the high efficacy of the diluted CPC solution may be that a biofilm susceptibility to CPC treatment depends on the amount of CPC bounded directly to the bacterial cell membrane and not on the total amount of bound CPC molecules, as shown by Caputo et al. [34]. CPC-EO, the solution where CPC was supplemented with EOs’ components, including eucalyptol, menthol, methyl salicylate and carvone, was the most effective against *S. aureus* biofilm comparing to other tested mouthrinses (Fig. 4A). The high antibiofilm potential was also observed in case of *L. rhamnosus* species and *Streptococcus* spp genus (Fig. 4D, E).These results may suggest some similarities in composition and structure of the biofilm formed by these species. The antimicrobial activity of EOs is strongly linked to their hydrophobicity. The cell walls of Gram-positive bacteria are made up predominantly of peptidoglycan, proteins or teichoic acids which do not create a barrier toward hydrophobic compounds such as those found in EOs [35]. Membrane/cell wall permeabilization can be related with alterations on their physicochemical properties caused by EOs through significant changes in the cellular surface charge, damage in cytoplasmic membrane and increased leakage of *K*^+^ [36]. Due to multidirectional mode of action, EOs display some kind of selectivity. It has been found that EOs are more active against pathogens than against beneficial bacteria [37]. The mechanism standing behind this phenomenon has not been fully elucidated, but it may constitute an assumption why CPC-EO was not as effective against the biofilm of *E. faecalis* or *C. albicans* as against other biofilms of the tested microorganisms (Fig. 4B; C) The other reason might be the fact that mixture of EOs components affect multiple biochemical processes in the bacteria, producing a plethora of interactive antibacterial effects that may contribute to biofilm disruption [38].

It should be also highlighted that both CPC and CPC-EO contain Zn^2+^ ions which enhance its' antimicrobial properties acting as a counterions and neutralizing negatively charged EPS components and, in consequence, mitigate the diffusion of pyridine ion through biofilm and reaching cell wall components [39]. This synergistic effect might be the indirect reason of high antibiofilm effectiveness of mouthrinse based on cetylpyridinium chloride.

The AgNP possesses antimicrobial activity against a wide spectrum of pathogens and not only prevents biofilm formation but it also kills bacteria in existing biofilms [40]. The level of *Streptococcus* spp. biofilm eradication was 37.28%, while in the case of other tested species did not exceed significance threshold (Fig. 6A). Many factors can influence antimicrobial activity of silver ions including its size and origin [41, 42]. In addition, it has been shown that the concentrations regarded as bactericidal are effective against planktonic cells but not biofilms. It is probably due to physicochemical properties of biofilm EPM, the complex architecture of biofilm structure, hindering the diffusion of silver particles electrostatic forces or a type of AgNP carrier which may favor adsorption and accumulation in biofilms and influence the diffusion [42, 43]. It should be highlighted that ozonated water is present in the tested AgNP formula. Some studies have shown its effectiveness against staphylococcal biofilms [

The antimicrobial activity of HY is based on the interaction between negatively charged sulfuric acid residues (SO_4_^2−^) and positively charged hydrogen atoms in water molecules. This leads to dehydration of a biofilm matrix which may contain up to 98% of water [46, 47]. In consequence, biofilm is denatured and destabilized and can then be removed by irrigation.

Biofilm structure (cells and matrix together) may be treated as colloidal hydrogel where so called ‘bound-water’, including pores and channels, plays a crucial role for its function [41]. It may be thus hypothesized, the more bound-water is adsorbed to biofilm’s EPM, the greater effectiveness of HY [48]. This compound displayed the highest ability to eradicate biofilm formed by *C. albicans, L. rhamnosus* and *S. aureus* (Fig. 6B) [49]. Obtained results may suggest that EPM of biofilms formed by *E. faecalis* and *Streptococcus* spp. may contain less bound-water comparing to biofilms of *C. albicans, L. rhamnosus* and *S. aureus*. Thus, dehydrating mechanism of action is arguably the preferred mechanism for combating streptococcal biofilms.

Among the substances present in coconut oil, such medium-chain fatty acids (MCFAs) as lauric acid and caprylic acid are responsible for the antimicrobial activity to the greatest extent. The killing effect may be caused by putative mechanisms, including disruption of glycolysis on the Embden-Meyerhof-Parnas pathway and direct impairment of cell wall as demonstrated on *S. aureus* species [50]. In coconut pulling simulation experiment, the highest degree of eradication was observed for the *C. albicans* biofilm (65.48%) (Fig. 6C). The mechanism of interaction between *Candida* spp. and coconut oil is not explained yet. Nevertheless, Thaweboon et al. has discovered that the *L. casei* strain was resistant to all tested oils, including coconut oil [51]. Also in our study *L. rhamnosus* exposed high resistance to coconut oil, however Rosenblat et al. observed high eradication of *C. albicans* biofilm during 60 min of contact time [52]. It can be thus assumed that the potency of the antibiofilm effect is proportional to the contact time and to the intrinsic properties of exposed strain. Due to the lipophilic nature of MCFAs and the small size of their molecules, these substances are able to penetrate effectively through biofilm structure and to bind to lipophilic components of cell walls and to break the integrity of these cellular structures [35, 50, 52].

For comparison purposes, biofilm eradication potential was measured with A.D.A.M method (Fig. 7). Out of the tested substances, the most potent and the least potent ones (indicated in previous tests performed in this study) in aspect of biofilm eradication potential, were selected. The results have shown that the effectiveness of an antimicrobial substance depends not only on the species which was applied against, but also on the strain-specific features within that species. A wide range of susceptibility patterns (understood as the differences in compound’s concentration able to eradicate biofilm) was mainly observed for *E. faecalis*, treated with CPC-EO formulation. This experiment also shows that the applied research setting significantly influences the obtained results. While in the A.D.A.M. test, the antibiofilm activity of AgNP was the highest one (among the tested oral hygiene measures), the average level of biofilm eradication, being result of CPC and PHMB application, was comparable (Fig. 7). Such a phenomenon may be caused by the fact that both biocides (although of different molecular characteristics) target the same site of microbial cell (namely their membranes) and disorganizes them, leading to cytoplasm outflow and cell’s death [31, 39]. From this perspective, the significantly lower antibiofilm activity of EO-CPC compared to CPC may be caused (or be one of the causative agents) by the competitive action of CPC and EO for the same target site (microbial membrane), as such an effect was already reported with regard to the EOs by other research teams [53].

Pictures taken with the SEM method confirmed biofilm formation on HA discs surface (Figs. 8 and 9). The complex structure of biofilm formed by *L. rhamnosus* and *S. mutans* was also observed in the control settings (Figs. 8A and 9A). In a field of view, a reduced number of biofilm-forming cells was observed after PHMB and CPC treatment (Figs. 8B,C and 9B,C). These observations have confirmed the legitimacy of all stages of our research and presented parametric analyses.

## Conclusion

In conclusion, the results have shown that tested biofilms showed a diversified sensitivity to the tested antimicrobials. This might be related to specific variances in the composition of the EPM and the functioning of microorganisms forming individual biofilms as well as contact time of biofilm with an antimicrobial agent. The highest antibiofilm activity for the widest range of the tested microorganisms was observed for cetylpyridine chloride while the lowest in case of chlorhexidine. However, neither mouthrinse formulation was able to completely wipe out tested biofilm. The antibiofilm activity of coconut oil shows high diversity depending on the species of a microorganism, against which the oil was used. Our findings here may relate to different types of oral biofilms and other biofilm-related phenomena. Further, obtained results may be easily implemented in clinical routine and improve dental plaque prophylaxis.

## Supplementary Information

**Additional file 1: Table 1.** All clinical strainsused in performed tests. **Figure 1.** Biofilm coated HA discs placed in agar wells - gingival pocketssimulation. **Table 2.** Ingredients of all tested commercial products. **Figure 2.** Biocellulose (BC) discs. **Figure 3.** Procedure diagram of the A.D.A.M. method A-cutting of agar tunnels, B - placing HA disks in agar tunnels, C – flooding HA discs with artificial saliva, D -covering tunnels with biocellulose saturated with tested solutions. **Figure 4.** Biofilm stained withTTC. A - *S. mitis*; B - *E.faecalis.* **Figure 5.**Two-species biofilm formed by *L.* *rhamnosus* and *S.* *mutans *(magnification 9 999x).

## Data Availability

All data generated or analysed during this study are included in this published article and its supplementary information files.

## Abbreviations

A.D.A.M: Antibiofilm Dressing's Activity Measurement
AgNP: Silver nanoparticles
ATCC: American Type Culture Collection
BHI: Brain Heart Infusion
CHX: Chlorhexidine
CPC: Cetylpyridine chloride
EO: Essential oils
EPM: Extracellular polymeric matrix
HA: Hydroxyapatite
HY: Sulphonated phenolics gel
MCFAs: Medium-chain fatty acids
MRS: Man, Rogosa, Sharpe
PCM: Polish Collection of Microorganisms
PHMB: Polyhexanide
SEM: Scanning electron microscope
TSB: Tryptone Soya Broth

## References

[1] Scannapieco FA, Dongari-Bagtzoglou A (2021). Dysbiosis revisited: Understanding the role of the oral microbiome in the pathogenesis of gingivitis and periodontitis: A critical assessment. J Periodontol.

[2] Sarkar P, Malik S, Laha S, Das S, Bunk S, Ray JG, Chatterjee R, Saha A (2021). Dysbiosis of Oral Microbiota During Oral Squamous Cell Carcinoma Development. Front Oncol.

[3] https://www.who.int/news-room/fact-sheets/detail/oral-health (Assessed on 25 Mar 2022)

[4] Peres MA, Macpherson LMD, Weyant RJ, Daly B, Venturelli R, Mathur MR, Listl S, Celeste RK, Guarnizo-Herreño CC, Kearns C, Benzian H, Allison P, Watt RG (2019). Oral diseases: a global public health challenge. Lancet.

[5] Kazeminia M, Abdi A, Shohaimi S, Jalali R, Vaisi-Raygani A, Salari N, Mohammadi M (2020). Dental caries in primary and permanent teeth in children's worldwide, 1995 to 2019: a systematic review and meta-analysis. Head Face Med.

[6] Editorial Board. The Lancet. 2009;373(9657):1. ISSN: 0140–6736. https://www.thelancet.com/journals/lancet/article/PIIS0140-6736(08)61933-9/fulltext. Assessed 25 Mar 2022.

[7] Sudhakara P, Gupta A, Bhardwaj A, Wilson A (2018). Oral Dysbiotic Communities and Their Implications in Systemic Diseases. Dentistry J.

[8] Marouf N, Cai W, Said KN, Daas H, Diab H, Chinta VR, Hssain AA, Nicolau B, Sanz M, Tamimi F (2021). Association between periodontitis and severity of COVID-19 infection: A case-control study. J Clin Periodontol.

[9] Marsh PD (2006). Dental plaque as a biofilm and a microbial community – implications for health and disease. BMC Oral Health..

[10] Hosseinpoor AR, Itani L, Petersen PE (2012). Socio-economic Inequality in Oral Healthcare Coverage: Results from the World Health Survey. J Dent Res.

[11] Winkelmann J, Gómez Rossi J, Schwendicke F (2022). Exploring variation of coverage and access to dental care for adults in 11 European countries: a vignette approach. BMC Oral Health..

[12] Vujicic M, Buchmueller T, Klein R (2016). Dental Care Presents The Highest Level Of Financial Barriers, Compared To Other Types Of Health Care Services. Health Aff (Millwood)..

[13] Takenaka S, Ohsumi T, Noiri Y (2019). Evidence-based strategy for dental biofilms: Current evidence of mouthwashes on dental biofilm and gingivitis. Jpn Dent Sci Rev..

[14] Woolley J, Gibbons T, Patel K, Sacco R (2020). The effect of oil pulling with coconut oil to improve dental hygiene and oral health: a systematic review. Heliyon.

[15] Maoyang Lu, Xuan S, Wang Z (2019). Oral microbiota: A new view of body health. Food Sci Human Wellness.

[16] Junka A, Szymczyk P, Ziółkowski G, Karuga-Kuzniewska E, Smutnicka D, Bil-Lula I, Bartoszewicz M, Mahabady S, Sedghizadeh PP (2017). Bad to the Bone: On In Vitro and Ex Vivo Microbial Biofilm Ability to Directly Destroy Colonized Bone Surfaces without Participation of Host Immunity or Osteoclastogenesis. PLoS ONE.

[17] Junka AF, Szymczyk P, Smutnicka D, Kos M, Smolina I, Bartoszewicz M, Chlebus E, Turniak M, Sedghizadeh PP (2015). Microbial biofilms are able to destroy hydroxyapatite in the absence of host immunity in vitro. J Oral Maxillofac Surg..

[18] Dydak K, Junka A, Dydak A, Brożyna M, Paleczny J, Fijalkowski K, Kubielas G, Aniołek O, Bartoszewicz M (2021). In Vitro Efficacy of Bacterial Cellulose Dressings Chemisorbed with Antiseptics against Biofilm Formed by Pathogens Isolated from Chronic Wounds. Int J Mol Sci.

[19] Junka AF, Żywicka A, Szymczyk P, Dziadas M, Bartoszewicz M, Fijałkowski K (2017). A.D.A.M. test (Antibiofilm Dressing's Activity Measurement) - Simple method for evaluating anti-biofilm activity of drug-saturated dressings against wound pathogens. J Microbiol Methods..

[20] Sakaue Y, Takenaka S, Ohsumi T (2018). The effect of chlorhexidine on dental calculus formation: an in vitro study. BMC Oral Health.

[21] Okshevsky M, Meyer RL (2015). The role of extracellular DNA in the establishment, maintenance and perpetuation of bacterial biofilms. Crit Rev Microbiol.

[22] Cieplik F, Jakubovics NS, Buchalla W, Maisch T, Hellwig E, Al-Ahmad A (2019). Resistance toward chlorhexidine in oral bacteria – is there cause for concern?. Front Microbiol.

[23] Pierce CG, Vila T, Romo JA, Montelongo-Jauregui D, Wall G, Ramasubramanian A, Lopez-Ribot JL (2017). The Candida albicans Biofilm Matrix: Composition, Structure and Function. J Fungi (Basel, Switzerland).

[24] Alvendal C, Mohanty S, Bohm-Starke N, Brauner A (2020). Anti-biofilm activity of chlorhexidine digluconate against Candida *albicans* vaginal isolates. PLoS ONE.

[25] Hutchings RL, Singh SM, Cabello-Villegas J, Mallela KM (2013). Effect of antimicrobial preservatives on partial protein unfolding and aggregation. J Pharm Sci.

[26] Hadhoum N, Guerfi B, Sider T, Yassa Z, Djerboua T, Boursouti M, Mamou M, Aoul F, Mekacher L (2018). 'Synthesis, Physicochemical Characterization and Study of the Antimicrobial Activity of Chlorobutanol'. World Academy of Science, Engineering and Technology, Open Science Index 143. Int J Pharmacol Pharm Sci.

[27] Ruiz L, Escribano C, Veiga-Crespo P, Villa TG (2007). In vitro comparative experimental study of antimicrobial action of mouth washing products. Bulletin du Girso..

[28] Babu JP, Garcia-Godoy F (2014). In vitro comparison of commercial oral rinses on bacterial adhesion and their detachment from biofilm formed on hydroxyapatite disks. Oral Health Prev Dent.

[29] Şahiner A, Halat E, Alğın YE (2019). Comparison of bactericidal and fungicidal efficacy of antiseptic formulations according to EN 13727 and EN 13624 standards. Turk J Med Sci.

[30] Machuca J, Lopez-Rojas R, Fernandez-Cuenca F, Pascual Á (2019). Comparative activity of a polyhexanide-betaine solution against biofilms produced by multidrug-resistant bacteria belonging to high-risk clones. J Hosp Infect.

[31] Sowlati-Hashjin S, Carbone P, Karttunen M (2020). Insights into the Polyhexamethylene Biguanide (PHMB) Mechanism of Action on Bacterial Membrane and DNA: A Molecular Dynamics Study. J Phys Chem B.

[32] Zheng Y, Wang D, Ma LZ (2021). Effect of Polyhexamethylene Biguanide in Combination with Undecylenamidopropyl Betaine or PslG on Biofilm Clearance. Int J Mol Sci.

[33] Latimer J, Munday JL, Buzza KM (2015). Antibacterial and anti-biofilm activity of mouthrinses containing cetylpyridinium chloride and sodium fluoride. BMC Microbiol..

[34] Caputo RA, Treick RW, Griffin CC, Farrell MP (1975). Rapid determination of the amount of cetylpyridinium chloride bound by bacteria. Appl Microbiol.

[35] Nazzaro F, Fratianni F, De Martino L, Coppola R, De Feo V (2013). Effect of Essential Oils on Pathogenic Bacteria. Pharmaceuticals.

[36] Lopez-Romero JC, González-Ríos H, Borges A, Simões M (2015). Antibacterial Effects and Mode of Action of Selected Essential Oils Components against Escherichia coli and Staphylococcus aureus. Evid Based Complement Altern Med.

[37] Ambrosio CMS, Ikeda NY, Miano AC, Saldaña E, Moreno AM, Stashenko E, Contreras-Castillo CJ, Da Gloria EM (2019). Unraveling the selective antibacterial activity and chemical composition of citrus essential oils. Sci Rep.

[38] Bassolé IH, Juliani HR (2012). Essential oils in combination and their antimicrobial properties. Molecules (Basel, Switzerland).

[39] Mao X, Auer DL, Buchalla W, Hiller KA, Maisch T, Hellwig E, Al-Ahmad A, Cieplik F (2020). Cetylpyridinium Chloride: Mechanism of Action, Antimicrobial Efficacy in Biofilms, and Potential Risks of Resistance. Antimicrob Agents Chemother.

[40] Martinez-Gutierrez F, Boegli L, Agostinho A, Sánchez EM, Bach H, Ruiz F, James G (2013). Anti-biofilm activity of silver nanoparticles against different microorganisms. Biofouling..

[41] Monowar T, Rahman MS, Bhore SJ, Raju G, Sathasivam KV (2018). Silver Nanoparticles Synthesized by Using the Endophytic Bacterium Pantoea ananatis are Promising Antimicrobial Agents against Multidrug Resistant Bacteria. Molecules.

[42] Rolim WR, Lamilla C, Pieretti JC, Diaz M, Tortella GR, Diez MC, Barrientos L, Seabra AB, Rubilar O (2019). Comparison of antibacterial and antibiofilm activities of biologically synthesized silver nanoparticles against several bacterial strains of medical interest. Energ Ecol Environ..

[43] Yin IX, Zhang J, Zhao IS, Mei ML, Li Q, Chu CH (2020). The Antibacterial Mechanism of Silver Nanoparticles and Its Application in Dentistry. Int J Nanomedicine.

[44] Bialoszewski D, Kalicinska A, Bocian E, Czajkowska M, Bukowska B, Tyski S, Pietruczuk-Padzik A (2011). Activity of ozonated water and ozone against Staphylococcus aureus and Pseudomonas aeruginosa biofilms. Med Sci Monit..

[45] Koyama R, Okuda K, Matsushita K, Beppu M, Mizunoe Y (2015). Antimicrobial and Antibiofilm Effects of Ozonated Water for Prevention and Treatment of Bone and Joint Infections. J St Marianna Univ.

[46] Pini-Prato G, Magnani C, Rotundo R (2016). Treatment of Acute Periodontal Abscesses Using the Biofilm Decontamination Approach: A Case Report Study. Int J Periodontics Restorative Dent..

[47] Ido N, Lybman A, Hayet S, Azulay DN, Ghrayeb M, Liddaweih S, Chai L. Bacillus subtilis biofilms characterized as hydrogels. Insights on water uptake and water binding in biofilms. Soft Matter. 2020;16:(26)6180–6190. 10.1039/d0sm00581a 10.1039/d0sm00581a 32567645

[48] Quan K, Hou J, Zhang Z, Ren Y, Peterson BW, Flemming HC, Mayer C, Busscher HJ, van der Mei HC. 2021. Water in bacterial biofilms: pores and channels, storage and transport functions. Critical reviews in microbiology, 1–20. Advance online publication. 10.1080/1040841X.2021.1962802 10.1080/1040841X.2021.1962802 34411498

[49] Karygianni L, Ren Z, Koo H, Thurnheer T (2020). Biofilm Matrixome: Extracellular Components in Structured Microbial Communities. Trends Microbiol.

[50] Widianingrum DC, Noviandi CT, Salasia SIO (2019). Antibacterial and immunomodulator activities of virgin coconut oil (VCO) against Staphylococcus aureus. Heliyon.

[51] Thaweboon S, Nakaparksin J, Thaweboon B (2011). Effect of oil-pulling on oral microorganisms in biofilm models. Asia J Public Health..

[52] Rosenblatt J, Reitzel RA, Vargas-Cruz N, Chaftari AM, Hachem R, Raad I (2017). Caprylic and Polygalacturonic Acid Combinations for Eradication of Microbial Organisms Embedded in Biofilm. Front Microbiol.

[53] Kowalczyk A, Przychodna M, Sopata S, Bodalska A, Fecka I (2020). Thymol and Thyme Essential Oil-New Insights into Selected Therapeutic Applications. Molecules.

