# Supplementary material for: The antibiofilm activity of selected substances used in oral health prophylaxis
Source: BMC Oral Health. 2022 Nov 17;22:509. doi: 10.1186/s12903-022-02532-4 (PMC9672622; doi:10.1186/s12903-022-02532-4)
Supplement: Supplementary file 1 — Additional file 1: Table 1. All clinical strainsused in performed tests. Figure 1. Biofilm coated HA discs placed in agar wells - gingival pocketssimulation. Table 2. Ingredients of all tested commercial products. Figure 2. Biocellulose (BC) discs. Figure 3. Procedure diagram of the A.D.A.M. method A-cutting of agar tunnels, B - placing HA disks in agar tunnels, C – flooding HA discs with artificial saliva, D -covering tunnels with biocellulose saturated with tested solutions. Figure 4. Biofilm stained withTTC. A - S. mitis; B - E.faecalis. Figure 5.Two-species biofilm formed by L. rhamnosus and S. mutans (magnification 9 999x). [file 12903_2022_2532_MOESM1_ESM.docx]

**The Antibiofilm Activity of Selected Substances Used in Oral Health Prophylaxis**

R. Dudek-Wicher^1*^, A.F. Junka^1*^, P.Migdał^2^ , A Korzeniowska-Kowal^3^, A. Wzorek^3^, M. Bartoszewicz^1^

^1^ Department of Pharmaceutical Microbiology and Parasitology, Faculty of Pharmacy, Medical University of Silesian Piasts in Wroclaw, 50-367 Wrocław, Poland; [r.dudek.wicher@gmail.com](mailto:r.dudek.wicher@gmail.com); [feliks.junka@gmail.com](mailto:feliks.junka@gmail.com); m.bartoszewicz@umw.edu.pl

^2^ Department of Environment, Hygiene and Animal Welfare, Faculty of Biology and Animal Science,

Wroclaw University of Environmental and Life Sciences, Chełmońskiego 38C, 51-630 Wrocław, Poland;

[pawel.migdal@upwr.edu.pl](mailto:pawel.migdal@upwr.edu.pl)

^3^ Polish Collection of Microorganisms (PCM), Department of Immunology of Infectious Diseases, Hirszfeld Institute

of Immunology and Experimental Therapy, Polish Academy of Sciences, Rudolfa Weigla 12, 53-114 Wroclaw, Poland

; [agnieszka.korzeniowska-kowal@hirszfeld.pl](mailto:agnieszka.korzeniowska-kowal@hirszfeld.pl), [anna.wzorek@hirszfeld.pl](mailto:anna.wzorek@hirszfeld.pl)

**Supplementary materials**

**Table 1:** All clinical strains used in performed tests

| **Lp.** | ***S. aureus*** | ***E. faecalis*** | ***L. rhamnosus*** | ***C. albicans*** | ***Streptococcus* spp*.*** |
| --- | --- | --- | --- | --- | --- |
| **Ref.** | *S. aureus* ATCC 6538 | *E. faecalis* ATCC 25212, | *L. rhamnosus* PCM 489, | *C. albicans* ATCC 10231 | *S. mutans* ATCC 25175; *S. sanguinis* ATCC 10556; *S. oralis PCM 2465; S. mitis PCM 2463* |
| **1** | 4648 | 54* | 4* | 9 | 3 |
| **2** | 508 | 55* | 11 | 11 | 8 |
| **3** | 2230 | 59 | 12 | 28 | 11 |
| **4** | 6755 | 60 | 21* | 34 | 16 |
| **5** | 8737 | 61 | 22 | 47* | 24 |
| **6** | 8983 | 734 | 23 | 50* | 26* |
| **7** | 5713 | 212 | 24 | 56* | 33 |
| **8** | 5489 | 214 | 26 | 67 | 36 |
| **9** | 1072 | 594 | 33* | 71 | 40* |
| **10** | 1815 | 610 | 34 | 79* | 41* |
| **11** | 4648 | 628 | 35* | 83 | 51 |
| **12** | MS1 | 700 | 36 | 84 | 52 |
| **13** | MS2* | 77141 | 37 | 86 | 54 |
| **14** | MS3* | 6768* | 38 | 89 | 55 |
| **15** | MS4* | 1863* | 39 | 94 | 64 |
| **16** | MS5* | 2836 | 40 | 97 | 65 |
| **17** | MS6 | 2852 | 42 | 98 | 69* |
| **18** | MS7 | 3076 | 43 | 106 | 70* |
| **19** | MS8 | 2849 | 53 | 117 | 73* |

***Strains selected to assessment of eradication potential of silver nanoparticles solution and coconut oil.**


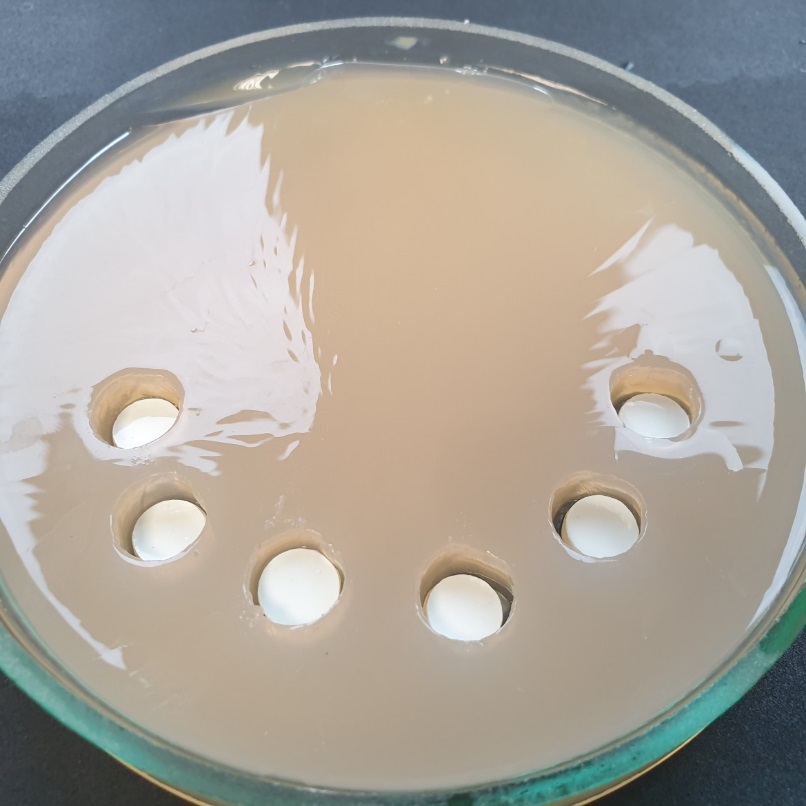


**Figure 1.** Biofilm coated HA discs placed in agar wells - gingival pockets simulation.

**Table 2**. Ingredients of all tested commercial products.

| **Symbol** | **Brand name** | **Ingredients** |
| --- | --- | --- |
| **CHX** | Eludril Classic; Pierre Fabre, Paris, France; LOT :G01453, Exp :05.2022 | Glycerin, Alcohol, Aqua, Flavor, CI 16255, **Chlorhexidine Digluconate**, Chlorobutanol, Diethylhexyl Sulfosuccinate, Limonene, Menthol. Chlorobutanol and Alcohol (44.2% v/v). |
| **PHMB** | Prontoral; Braun, Frankfurt, Germany; LOT:2122M13, Exp:02.2022 | Aqua, PEG-40 Hardened Castor Oil, Flavor, Sodium Cyclamate, Undecylenamide Dopropyl Betaine, **Polyaminpropyl (Polyhexanide)**. |
| **CPC** | Vitis Gingival; Dentaid, Gdansk, Poland; LOT: P1004, Exp: 01.2022 | Aqua, Propylene Glycol, Glycerin,  Xylitol, Sodium Gluconate, Panthenol, Poloxamer 407, Sodium Lactate, Sodium Benzoate, Zinc Lactate, Allantoin, Sodium Methylparaben, Lactic Acid, **Cetylpyridinium chloride**, Sodium Saccharin, Aroma, Cl 16035. |
| **CPC/EO** | Bactericin Pro; Primer Sp. z o.o., Bojszowy-Swierczyniec, Poland; LOT:103MB12, Exp.03.2022 | Aqua, Glycerin, Glycol, Sodium Citrate, E**ucalyptol, Rona Care, Menthol, Methyl Salicylate, Carvone,** Dipotassium Hydrogen Phosphate, HCL, Zinc Chloride. |
| **AgNP** | Parosin; Arkona, Niemce, Poland; LOT:20200901, Exp: 08.2022 | Ozonated Water, **Silver Nanocolloid,** Sodium Nitrate, Orthophosphoric Acid, Lactic Acid, Aromas. |
| **HY** | Hyben X; EPIEN Medical, Inc., St. Paul, MN, USA; LOT: P20S62, Exp.09.2022 | **Sulphonated phenolics**, sulfuric acid, Aqua, Colloidal silica, Allura Red AC. |

**
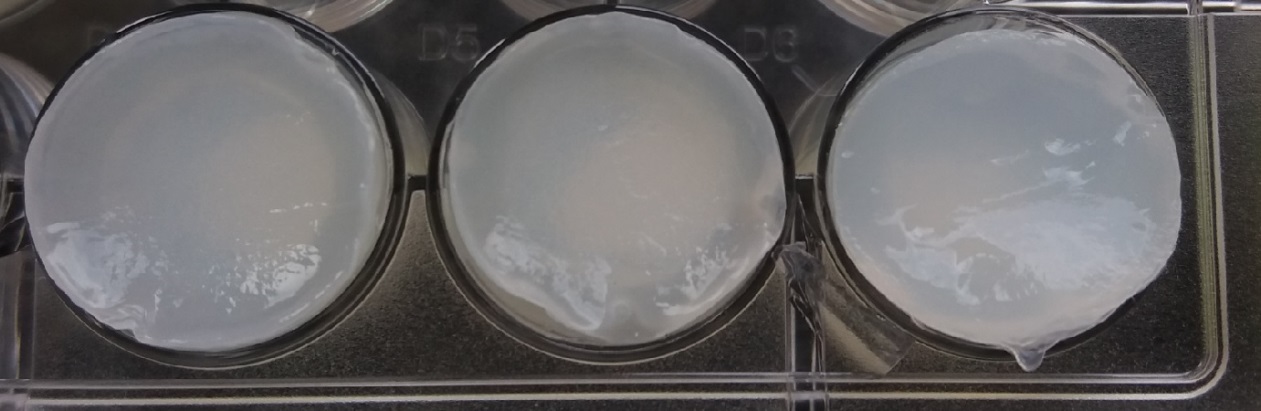
**

**Figure 2.** Biocellulose (BC) discs.


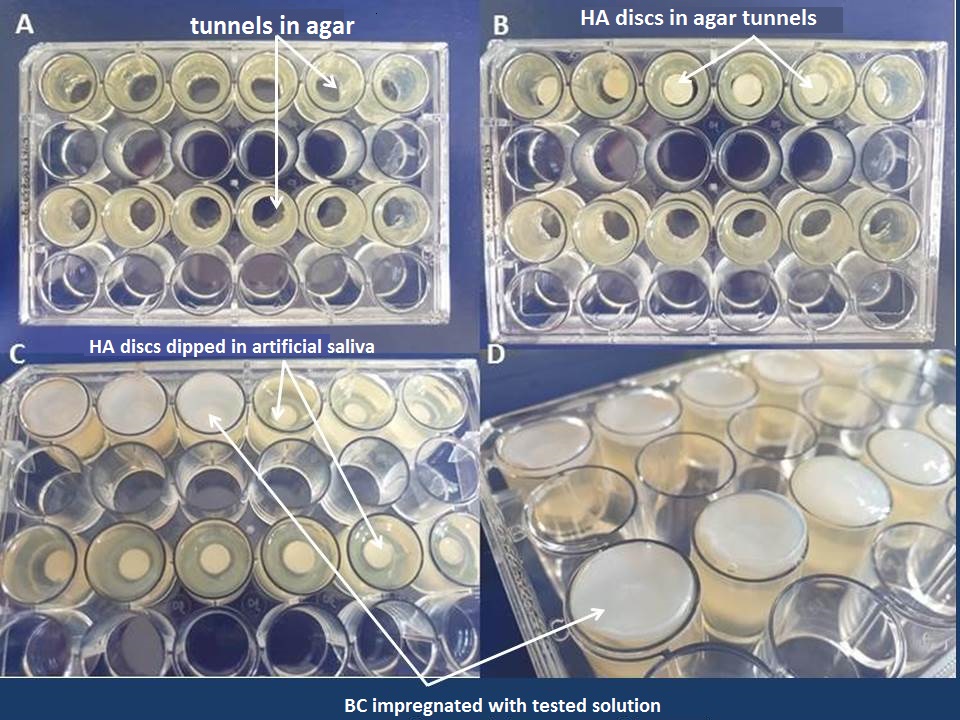


**Figure 3.** Procedure diagram of the A.D.A.M. method A- cutting of agar tunnels, B - placing HA disks in agar tunnels, C – flooding HA discs with artificial saliva, D - covering tunnels with biocellulose saturated with tested solutions.

**
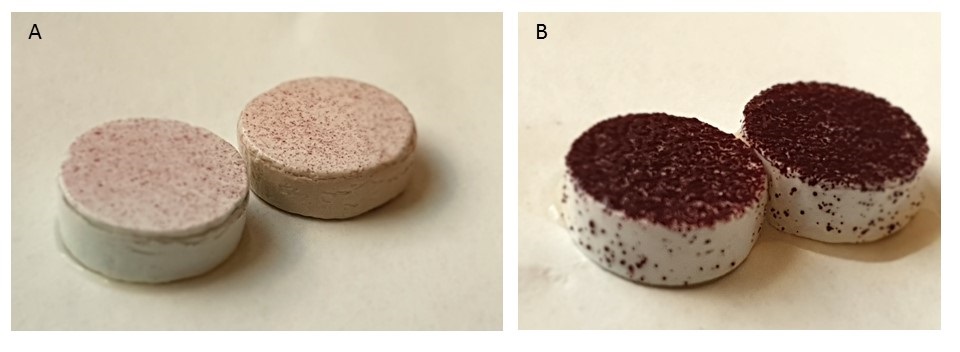
**

**Figure 4.** Biofilm stained with TTC. A - *S. mitis*; B - *E. faecalis.*

**
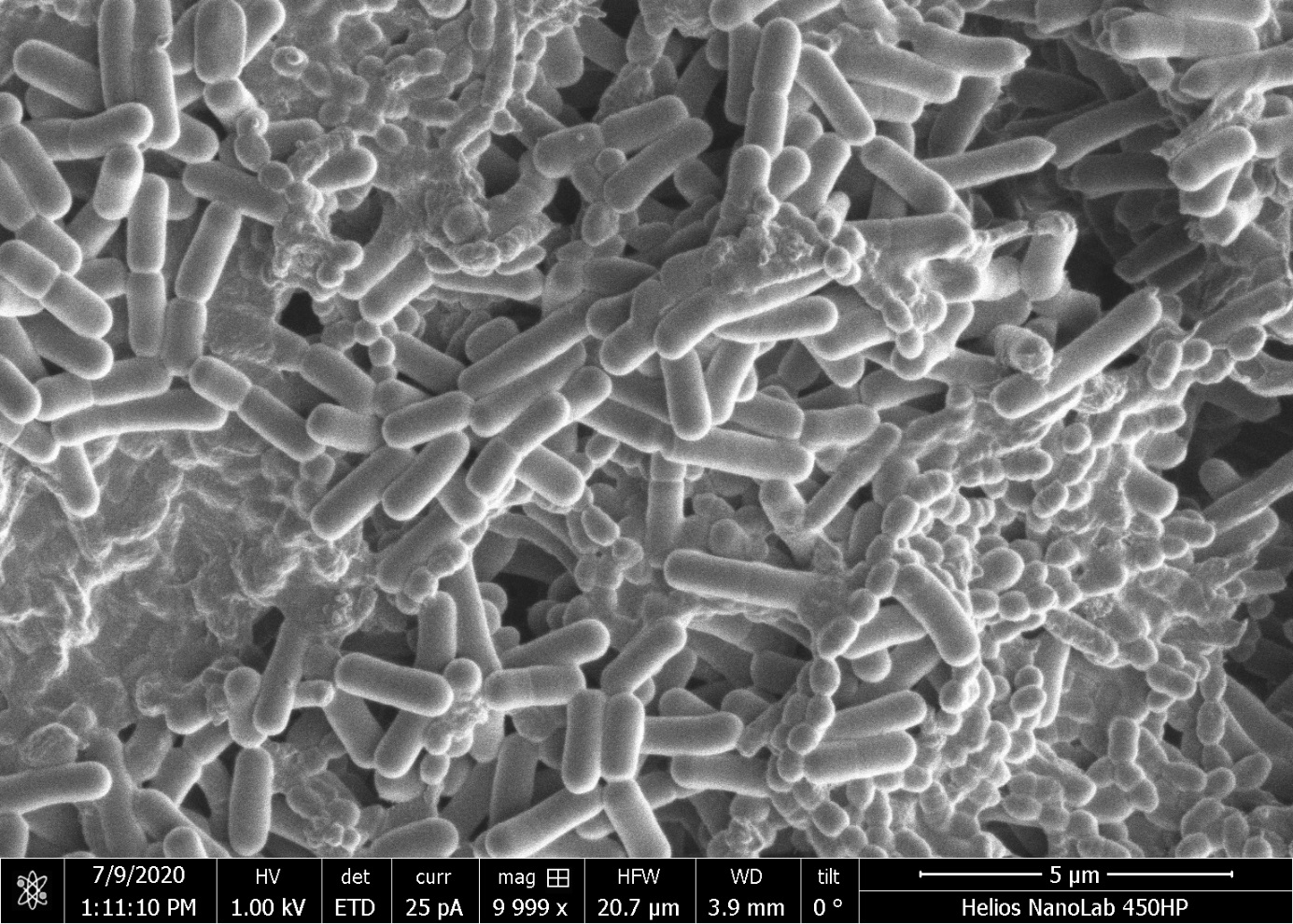
Figure 5.** Two-species biofilm formed by *L. rhamnosus* and *S. mutans* (magnification 9 999x).
